# Re-designing the pathway to surgery: better care and added value

**DOI:** 10.1186/s13741-017-0065-4

**Published:** 2017-06-20

**Authors:** Michael P. W. Grocott, James O. M. Plumb, Mark Edwards, Imogen Fecher-Jones, Denny Z. H. Levett

**Affiliations:** 1 Anaesthesia and Critical Care Research Unit, University Hospital Southampton NHS Foundation Trust, Southampton, UK; 2 Critical Care Research Area, NIHR Respiratory Biomedical Research Unit, University Hospital Southampton NHS Foundation Trust, Southampton, UK; 3 Integrative Physiology and Critical Illness Group, Clinical and Experimental Sciences, Faculty of Medicine, University of Southampton, Southampton, UK; 4 GICU, Mailpoint 27, D Level, Centre Block, University Hospital Southampton NHS Foundation Trust, Tremona Road, Southampton, Hampshire SO16 6YD UK; 5 Department of Anaesthesia, E Level, Centre Block, University Hospital Southampton NHS Foundation Trust, Tremona Road, Southampton, Hampshire SO16 6YD UK

**Keywords:** Perioperative medicine, Surgery, Anaesthesia, Surgical pathway, Re-design, Surgery school, Business process re-engineering, Collaborative decision-making, Comorbidity management, Collaborative behavioural change: “lifestyle modification”

## Abstract

The case for radical pathway re-design before surgery is in part driven by healthcare system pressures which are in turn the result of continuously rising demand in the face of tightly constrained resources. Such circumstances tend to drive revolutionary, rather than incremental, change. The current approach to preoperative assessment, that typically occurs in the weeks leading up to surgery, but is all too often only a few days before surgery, results in a lost opportunity for perioperative physicians to improve patient care. Re-engineering this process based on a patient-focused, pathway-driven vision of perioperative medicine offers a means of exploiting this opportunity. This review explores drivers for change, the opportunity offered by pathway re-design, and suggests a variety of strategies to add value in the preoperative pathway, each of which is facilitated by early engagement between perioperative physician and patient: collaborative decision-making, collaborative behavioural change, targeted comorbidity management as well as expectation management and psychological preparation for surgery including surgery schools.

## Introduction

Contemporary healthcare is characterized by severe resource constraints in the face of unremitting increases in demand, in large-part due to demographic change. The 2007–2008 global economic crisis and the subsequent Great Recession of 2008–2012 resulted in fiscal tightening that in turn led to flat or falling healthcare spending, expressed as a proportion of Gross Domestic Product, in many countries (Health and care funding in a nutshell | the health foundation & Available from: http://www.health.org.uk/health-and-social-care-funding-explained#Future. Accessed 20 Dec 2016). At the same time, improvements in public health and medical care have led to increased life expectancy, but declines in mortality have not been matched by similar declines in morbidity; people are living longer with disease and the prevalence of multi-morbidity is rising (Newton et al. 2015; Barnett et al. 2012). Continuing healthcare innovation drives an additional and sustained cost pressure within healthcare budgets (Health and care funding in a nutshell | the health foundation & Available from: http://www.health.org.uk/health-and-social-care-funding-explained#Future. Accessed 20 Dec 2016). This progressive increase in burden in the context of tightly constrained resourcing is only sustainable through either degradation of service quality and/or scope, or radical improvements in efficiency and cost-effectiveness. In this context, slow incremental change may not provide credible solutions. Radical process re-design, based on a fundamental re-evaluation of goals from a patient perspective, may be needed: revolution, not evolution, may be the answer to this existential challenge.

## Process re-engineering

The Institute of Health Improvement in the USA places healthcare cost reduction as a key dimension of the “Triple Aim” framework for optimizing health system performance and population health benefit (Stiefel & Nolan 2012). In modern healthcare, where resources are inevitably constrained, it is incumbent on clinicians seeking to change practice to demonstrate not only that a new approach improves clinical quality but also that it is of good value, defined by the relationship between cost and clinical effectiveness (Grocott & Mythen 2015). So how can anaesthetists and perioperative physicians add value to our patient’s pathway to surgery?

The concept of business process re-engineering (BPR) is commonly attributed to the 1990 Harvard Business Review article by Michael Harmer entitled “Reengineering work: don’t automate, obliterate” (Hammer 1990). Perhaps the most concise definition of this business management strategy is: “… the fundamental rethinking and radical re-design of business processes to achieve dramatic improvements in critical contemporary modern measures of performance, such as cost, quality, service, and speed” (Hammer & Champy 1993). BPR focuses on radical change, rather than continuous iterative improvement, and was introduced in the context of increasing use of information technology (IT) in industry. BPR sought to rethink the application of such technology, away from using IT to replace existing roles, towards fundamental system re-design, based on a comprehensive re-evaluation of process aims. Is it time to apply such thinking to the pathway our patients take towards surgery?

## Value and perioperative medicine

Perioperative medicine is defined as the practice of patient-centered, multidisciplinary, and integrated medical care of patients from the moment of contemplation of surgery until full recovery (Grocott & Mythen 2015). Explicit to this definition is the patient and their pathway of care as the central focus. In contrast, traditional care models have tended to define themselves by “silos” of delivered care (e.g. operating room suite) or professional activity (e.g. administering anaesthetics). Such silo-based thinking is antithetical to modern concepts of improving value in healthcare, which emphasize the importance of considering value across the whole patient pathway. In the words of Michael Porter, the business thinker turned healthcare reform advocate “The proper unit for measuring value should encompass all services or activities that jointly determine success in meeting a set of patient needs.” (Porter 2009).

## The pathway to surgery

**Fig. 1:**
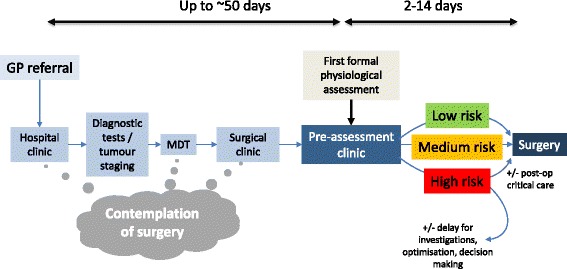
Traditional model of the journey from GP referral through to surgery

For the patient, the pathway to surgery commences when they first contemplate surgery in primary care or a surgical clinic and culminates weeks to months later on the day of surgery. In most settings, anaesthetic engagement with patients prior to surgery commences, at best, with preoperative assessment. This typically occurs in the weeks leading up to surgery, however, is all too often only a few days before surgery (Fig. 1), and at worst does not occur at all. Consequently, any contribution perioperative physicians might have to improving patient preparation for surgery is profoundly limited by one key factor: the limited time available between meeting the patient for the first time and the date of surgery. This in turn minimizes anaesthetists’ capacity to provide patient benefit. Opportunities to optimize therapy or behaviours have been missed and by this time patient expectations about their approaching surgery are long established and firmly set. So why do perioperative physicians not engage with their patients earlier?

**Fig. 2:**
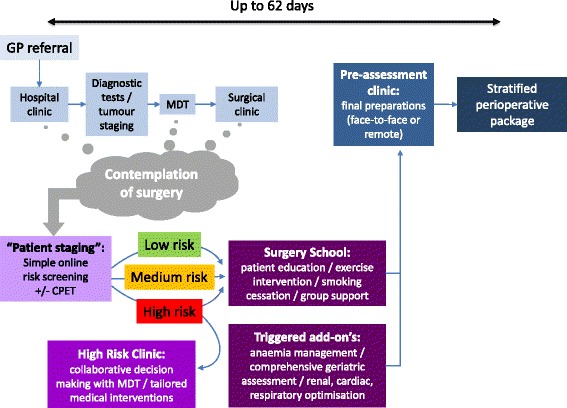
The pathway “re-engineered”—a model of process evolution in perioperative pathways. This re-engineered model aims to shift the timing of pre-assessment to much earlier in the pathway using simple online risk stratification tools and then early staging using objective physiological assessments (namely CPET). The aim is to have detailed information prior to any decision to operate with true collaborative decision-making taking centre stage. Surgery school and any “bolt-ons” occur in conjunction aiming to have things optimized prior to final decisions regarding surgery

The preoperative pathway has two key functions: to ensure that the right decision is made in relation to the surgery and to ensure that the patient is as well prepared as possible in order to maximize their resilience to the physiological stress of surgery. Both of these functions are more readily achieved when the perioperative physician has the opportunity to engage with their patient earlier in the pathway to surgery (Fig. 2). Early engagement with patients, as soon as possible after the moment of contemplation of surgery, is probably best achieved through screening questionnaires administered during the first discussion of the possibility of surgery. Such engagement may occur at the time of initial diagnosis of pathology (e.g. cystoscopy clinic, colonoscopy clinic) or at the point where the patient chooses to contact a physician to discuss troubling symptoms (e.g. hip pain consultation in a GP practice). The content of these questionnaires might encompass basic demographic data, items to screen for comorbidities (e.g. diabetes, heart and lung disease) as well as questions about relevant behaviours (e.g. cigarettes, alcohol, activity and diet). The nature of these questionnaires may vary between settings, but substantial opportunities exist for the application of technological solutions, such as tablet or mobile telephone-based applications, in order to facilitate the rapid transmission of data to the perioperative care team. Patients may then be triaged by risk category to low-, medium- and high-risk pathways. Patients estimated to have a high risk of adverse outcome may benefit from early referral to specialist “high-risk” clinics and advanced investigations such as cardiopulmonary exercise testing (Levett & Grocott 2015). Patients estimated to have a medium-level risk of adverse outcome might follow a more conventional pathway, with additional educational opportunities afforded by “surgery schools” (see below). Patients estimated to have a low risk of adverse outcome might bypass pre-assessment arrangements entirely and simply be offered “surgery school” (Trust tests ‘surgery school’ to get patients fit for ops | news | nursing times & Available from: https://www.nursingtimes.net/news/research-and-innovation/trust-tests-surgery-school-to-get-patients-fit-for-ops/7011466.article. Accessed 20 Dec 2016; Surgery school for patients | medicine | university of southampton & Available from: http://www.southampton.ac.uk/medicine/news/2016/10/surgery-school-to-get-patients-fit.page. Accessed 20 Dec 2016; Your surgery 3.Pdf, Available from: https://www.cmft.nhs.uk/media/1461701/your%20surgery.pdf. Accessed 20 Dec 2016). Surgery schools are multidisciplinary team (MDT)-delivered, predominantly classroom-based, interactive learning environments where patients come together as a group to learn more about their surgical journey and how they can improve it. A similar concept is seen in “antenatal classes” for expectant parents. Patients have the opportunity of meeting other patients going through the same journey along with members of the MDT and professionals involved with smoking/alcohol cessation, exercise and weight loss. Our experience of running such a school is that patients report benefit in the social, psychological and physiological domains.

## Opportunities to add value before surgery

Early engagement with patients before surgery, as soon as possible after the defining “moment of contemplation of surgery”, opens up many opportunities to improve the chances of meeting the twin aims of optimizing decision-making and maximizing resilience (see Table 1 and Fig. 2). Whilst surgeons may be expert in the prognosis estimates relating to the procedure type, anaesthetist/perioperative physicians may be better informed about the risks relating to functional status, chronic health and acute physiological deterioration. Risk factors for adverse outcome following surgery may be divided into fixed (e.g. age, gender) and modifiable (e.g. anaemia, physical fitness) (Table 1). Fixed and modifiable risk factors may be addressed during collaborative decision-making. Modifiable factors may be amenable to optimization through collaborative behavioural change and/or targeted comorbidity management (Table 1).

**Table 1:** Opportunities presented by earlier preoperative patient engagement

1.	Collaborative (shared) decision-making
2.	Collaborative behavioural change: “lifestyle modification”
3.	Comorbidity management
4.	Expectation management and psychological preparedness

### Collaborative (shared) decision-making

The evaluation of likely benefits and harms of surgery can proceed in parallel so that at the time of decision-making, patients may weigh the competing factors together and come to a well informed decision. Benefits from surgery derive almost exclusively from the primary therapeutic aim of the procedure (e.g. tumour removal). Harms from surgery derive in general from the unintended consequences of the necessary processes of perioperative and surgical care (e.g. tissue trauma, hypovolaemia, starvation). Benefits to the patient are characterized through careful evaluation of the primary pathology and the planned surgical procedure, for example through precision imaging of a tumour. Harms to the patient are best characterized by evaluating physiological resilience to the predictable pathophysiological challenges encountered around the time of surgery. Patients can only truly weigh these factors if they understand the outcome benefits associated with good “surgical outcome”, the spectrum of harms associated with limited physiological resilience, and how these factors interact with the context of their own life. Collaborative, or shared, decision-making provides the practice framework for achieving this goal (Glance et al. 2014). Value is added through reducing the incidence of “wrong patient surgery” (demand management) and as a consequence of the nature of the patients who choose to decline surgery. Those patients most likely to decide not to have surgery *may* well be those at greatest risk of complications following surgery. This is because those patients who are aware that they are at high risk for adverse outcome, and therefore where the harms of surgery may outweigh any benefit, are those patients most likely on balance to be those who decide against surgery. This is unlikely to be because patients intrinsically know their risk level, but rather is a product of full and effective communication of the harms and benefits of surgery. Patients who have complications following surgery incur costs that are two- to threefold higher than patients who avoid postoperative complications (Pradarelli et al. 2016; Birkmeyer et al. 2012; Vonlanthen et al. 2011). There are many other unrelated reasons why patients may decline surgery, which on occasions may seem unusual to the physician, but which make sense in the context of their personal beliefs and preferences. Exploring these beliefs and preferences and the options available in a collaborative manner enables a truly informed choice for the patient.

### Collaborative behavioural change

Collaborative behavioural change offers the opportunity to beneficially modify patient risk profiles and increase resilience prior to surgery. Smoking, alcohol consumption, diet and physical activity levels, the so-called lifestyle factors, are all linked to outcome following major surgery. More importantly, each of these factors is amendable to modification through behavioural change on a timeframe that can be achieved between the moment of contemplation of surgery and the procedure itself (Levett & Grocott 2015). Whilst improving any of these factors might offer a health benefit at any time in a patient’s life, there is a good argument that the period of time immediately before surgery offers a unique “teachable moment” when patients are particularly susceptible to positive behavioural messaging. The emerging science of behavioural economics aims to “meet patients half way”. Recognizing that patients do not always make decisions that optimize their own welfare, the aim of the this approach is to encourage change within existing patterns of behaviour, rather than asking patients to modify their behaviour to something more health promoting (Volpp & Asch 2016). Value is added through reducing postoperative complications and therefore cost as well the possibility of long-term behavioural change and public health benefit. In order for this value proposition to be viable, the aggregate costs of the intervention should be less than the resulting aggregate cost reduction. The costs of such interventions range from minimal (e.g. simple advice) and largely ineffective, to more expensive (e.g. structured in-hospital exercise training) with improved effectiveness (West et al. 2015). The public health benefits of long-term behavioural change, sustained long after the immediate perioperative episode is completed, are more difficult to characterize but may still be important. For example, smoking cessation is well documented to improve outcomes in a range of specialties and the “teachable moment” that comes with major surgery has been shown to improve a wide range of outcomes (Goltsman et al. 2017; Er Dedekargınoğlu et al. 2016; Jackson & Devine 2016).

### Comorbidity management

When identified and characterized early in the pre-surgical pathway, many comorbidities are amenable to management that can improve the patients physiological resilience to the surgical episode and thereby improve outcome following surgery. Specialist preoperative clinics delivered collaboratively by anaesthetists/perioperative physicians and relevant specialists are increasingly offering this service before surgery (e.g. perioperative anaemia clinics) (Guinn et al. 2016). Often these are virtual clinics, running efficiently without the requirement for patients to attend a physical place unless specific hospital-delivered therapy (e.g. intravenous iron) is required. Value may be added through reduced complications leading to improved outcomes and thereby reducing costs, and from more proximate measures. For example, the reduction in transfusion costs resulting from anaemia management may offset or even exceed the costs of an anaemia clinic (Evans et al. 2017; Froesslar et al. 2017; Froessler et al. 2016). In a recent study from Germany, economic modeling showed savings of €785.54 per anaemic patient treated with intravenous iron undergoing elective abdominal surgery due to a combination of reduction in blood transfusion and length of stay (Froesslar et al. 2017).

### Psychological preparation for surgery

Finally, patients can be better prepared psychologically for surgery. Patient information, helping to manage expectations, has long been considered a key element of enhanced recovery pathways (ERPs) (Grocott et al. 2012) and has traditionally been delivered in surgical and anaesthetic pre-assessment clinics. More sophisticated psychological preparation for surgery, for example using cognitive behavioural education, may provide additional benefit (Rolving et al. 2016). As we dissect the functions of the various elements of the pathway to surgery it becomes clear that diagnosis, prognosis and shared decision-making do not have to be undertaken at the same time and place as preparation for surgery. Increasingly, the concept of a separate classroom-based “Surgery School” is being pursued as a group patient activity (Trust tests ‘surgery school’ to get patients fit for ops | news | nursing times & Available from: https://www.nursingtimes.net/news/research-and-innovation/trust-tests-surgery-school-to-get-patients-fit-for-ops/7011466.article. Accessed 20 Dec 2016; Surgery school for patients | medicine | university of southampton & Available from: http://www.southampton.ac.uk/medicine/news/2016/10/surgery-school-to-get-patients-fit.page. Accessed 20 Dec 2016; Your surgery 3.Pdf, Available from: https://www.cmft.nhs.uk/media/1461701/your%20surgery.pdf. Accessed 20 Dec 2016). Value is added through driving behavioural change as well as through effectively preparing patients for early mobilization, eating and drinking after surgery.

## Conclusions

Re-designing the pathway to surgery so that perioperative physicians encounter their patients earlier in the perioperative journey opens up many opportunities to improve patient care. Collaborative decision-making offers the means of ensuring that each patient makes the right decision about which treatment option they wish to choose, including surgery. Collaborative behavioural change offers a route to improving modifiable behavioural characteristics prior to surgery through active programmes of alcohol cessation, smoking cessation, activity/exercise and dietary intervention. Surgery schools offer the opportunity to share such knowledge with all patients and thereby guide them towards healthier behaviours. Surgery schools also offer the opportunity to manage expectations in relation to the in-hospital surgical journey and improve psychological preparation for surgery. Each of these interventions offers a particular value proposition based on their relative costs and outcome benefits. Together, these interventions offer an opportunity to optimize patient decision-making in relation to surgical interventions and to maximize our patients resilience to the physiological stress of surgery through targeted management of modifiable risk factors.

## Abbreviations

BPR: Business process re-engineering
CPET: Cardiopulmonary Exercise Testing
ERP: Enhanced recovery pathways
IT: Information technology
MDT: Multidisciplinary team
